# Screening for Neurodevelopmental Delay for Preterm Very Low Birth Weight Infants at Tertiary Care Center in Saudi Arabia

**DOI:** 10.7759/cureus.20092

**Published:** 2021-12-01

**Authors:** Mohammed Y Al-Hindi, Bashaer H Almahdi, Dinah A Alasmari, Raghad K Alwagdani, Wujud M Hunjur, Abdullah F Khalel, Mansour A AlQurashi

**Affiliations:** 1 College of Medicine, King Saud Bin Abdulaziz University for Health Sciences, Jeddah, SAU; 2 Research Office, King Abdullah International Medical Research Center, Jeddah, SAU; 3 Department of Pediatrics, Neonatology Division, King Abdulaziz Medical City, Ministry of National Guard Health Affairs, Western Region, Jeddah, SAU; 4 Department of Pediatrics, King Abdulaziz Medical City, Ministry of National Guard Health Affairs, Western Region, Jeddah, SAU

**Keywords:** saudi arabia, ages and stages questionnaire third edition – arabic version, very low birth weight, neurodevelopment outcome, preterm premature

## Abstract

Background

Preterm infants are more susceptible to death, short-term complications, and long-term complications such as neurodevelopmental impairments. However, definitive assessment tools are not available in a resource-limited setting. Hence a screening tool is needed the Arabic-speaking population.

Method

Infants born at a gestational age of <32 weeks or a very low birth weight (VLBW) of less than 1500 g were recruited into a cross-sectional study. We identified infants (n = 61) admitted to the neonatal ICU at King Abdulaziz Medical City and reached 18 up to 24 months of corrected gestational age (CGA). The developmental assessment was done at 18, 20, 22, and 24 CGAs using the Ages and Stages Questionnaire third edition - Arabic version (ASQ3-A). The primary outcomes are early detection rate of neurodevelopmental delay (NDD), defined as a delay in one or more of the following: communication, gross motor, fine motor, problem-solving, and personal-social skills as per ASQ3-A.

Results

Sixty-one out of 92 eligible infants (36 excluded) completed the sufficient assessment. Twenty-six infants (42.6%) had at least one NDD in one of the following domains: communication skills: (11.5%), gross motor: (11.5%), fine motor: (19.7%), problem-solving skills: twelve infants (19.7%), and personal-social skills: twenty infants (23%). Perinatal events and periventricular leukomalacia (PVL) were significant independent predictors for the NDD.

Conclusion

This single-center study in Saudi Arabia screened preterm, VLBW infants based on ASQ3-A, twenty infants (42.6%) had an abnormal NDD at a corrected age of 18-24 months. Perinatal events and PVL were independent predictors of NDD. We recommend that all preterm VLBW infants in Saudi Arabia be evaluated by a neurodevelopmental screening tool, ASQ3-A, especially in resource-limited settings to start early intervention. Also, more extensive multicenter studies are to be carried out with definitive diagnostic tools to have a national benchmark for the long-term neurodevelopmental impairment.

## Introduction

The WHO reported that 15 million babies are born prematurely, born before 37 weeks, worldwide every year [1]. Preterm is classified into three subcategories, moderate or late preterm (from 32 to less than 37 weeks), very preterm (from 28 to 32 weeks), and extremely preterm, which is <28 weeks [1-4]. Moreover, they are born with very low birth weight (VLBW), under 1,500 gm, or extremely low birth weight (ELBW), below 1,000 gm [5-7]. Preterm births and VLBW have been associated with risk factors such as maternal infections, multiple pregnancies, genetic factors, and chronic conditions such as hypertension and diabetes [1,8]. Death is the most severe outcome, which is inversely proportional to the infants' gestational age and birth weights [3,9]. This approximately accounts for 1 million premature deaths each year [1]. As more preterm infants survive, they are more susceptible to short-term morbidities acquired during their stay in neonatal intensive care units (NICU) and long-term complications [9,10]. Such maternal or short-term morbidities may affect the long-term complications, namely neurodevelopmental impairments (NDI), which may have lifelong impacts on preterm infants [3,10-12]. Neurodevelopment, defined as the development of the CNS and the neurological processes, affects tasking and normal performance like gross motor development [13,14]. Thus, NDI mainly affect vision, hearing, social skills, learning ability, behaviors, and other aspects of life [15]. Such impairments might be aggravated by developmentally supportive therapies access, socioeconomic status of the family, and lifelong complications that necessitate feeding tubes and oxygen supplementation [3,16].

The routine neurodevelopmental assessment for preterm VLBW infants is carried out around the world. It includes multidisciplinary, longitudinal, and extensive evaluation which is the Bayley Scales of Infant Development [17]. It is costly, lengthy, and requires trained personnel; hence, the Ages and Stages Questionnaire, version 3 (ASQ3), is a useful alternative tool that can effectively screen developmental delay in limited-resource settings [18]. It is quick, inexpensive, easy to administer and interpret, and is accepted by the American Academy of Pediatrics as a valid developmental screener tool [19]. A new Arabic version was developed (ASQ3-A) and being used [20].

There is a lack of studies that have been published in Saudi Arabia on monitoring preterm infants and conducting their long-term neurodevelopmental outcomes. Moreover, research is required to develop standardized values for health care workers to understand the local incidence of neurodevelopmental delay (NDD) among preterm children. This study aims to measure the rates for NDD in infants born preterm, VLBW using the ASQ3-A tool in a local tertiary care center in Saudi Arabia. In addition, this research will provide neurodevelopmental values that can assist parents with preterm infants in perinatal counseling and assist health care workers.

## Materials and methods

Method

This study is a cross-sectional design conducted at King Abdulaziz Medical City (KAMC), Jeddah, Saudi Arabia, seen between June 1, 2019 and December 30, 2020. It included all infants born at the gestation of <32 weeks or VLBW, admitted to NICU at the KAMC, and reached the age of 18 to 24 months corrected gestational age (CGA). Infants who had documented chromosomal abnormalities, significant congenital anomalies, inherited inborn errors of metabolism, or severe perinatal asphyxia, were excluded from the study.

The specific objectives were to assess the applicability of the Ages and Stages Questionnaire (ASQ3-A) to early NDD, defined as a score of less than one SD (1SD) of one or more of the following: communication, gross motor, fine motor, problem-solving, and personal-social domains as per ASQ3-A scoring. The screen of NDD is carried out at 18, 20, 22, and 24 months CGA in the respective cohort. The secondary objective was to examine for possible confounding factors to neurodevelopmental outcomes. The patients were identified from the NICU logbook of admission, and the maternal and infant data were extracted from the electronic health records (BESTCare). Consents were obtained from one of the legal guardians prior to data collection. The study was approved by the local IRB at King Abdullah International Medical Research Center with a reference number SP18/189/J.

Data collection

The involved staff was trained to administer the ASQ3-A to help parents fill up the questionnaire and answer any queries. A senior specialist and a neonatologist supervised them. We interviewed the parents to collect developmental milestone changes as per the ASQ3-A. ASQ3-A is a questionnaire that is used effectively to screen any developmental delay in the first five and half years of a child's life. The five domains of the ASQ3-A developmental screening areas are communication, problem-solving, fine motor, gross motor, and personal-social. The ASQ3-A was divided into two parts. The first part was done by interviewing the parents via phone call, and the second part was done through an online survey after parents observed fine motor and problem-solving skills. After completing the questionnaires, the scores were recorded. Then, raw data was entered into the data collection sheet.

ASQ3-A is a valid and simple screening tool used confidently to distinguish and exclude neurodevelopmental impairment as its sensitivity, and the negative predictive value approaching 100%, its specificity was acceptable (76%) [21]. However, sensitivity to detect infants with impairments ranged from 20 to 60% [22]. ASQ3-A is a suggested screening tool in routine monitoring of low-risk children due to its psychometric properties. Also, it is recommended to use ASQ3-A in the follow-up programs for preterm infants and children who have biological risk factors [23]. ASQ3-A was translated and verified by experts from different Arabic-speaking countries, including Saudi Arabia, Egypt, and Kuwait, to assure that the translation is accurate and accessible [24].

Data analysis

Data were entered into SPSS version 27 or SATA version 15.1. Frequency, percentages, and bar charts were used to represent the qualitative data. The quantitative data were described using the mean and SD or median and quartiles as per normality. The reason why we defined NDD as a score of less than 1SD of one or more of the five domains of ASQ3-A is to detect more premature children with delays as suggested by Simard MN et al. [22]. For univariate analysis, we used Chi-squared, Fisher's Exact, or t-test accordingly. We used Firth's logistic regression as a standard approach for analyzing binary outcomes with small samples to assess for significant predictors. Whereas it reduces the bias in maximum likelihood estimates of coefficients, bias towards one-half is introduced in the predicted probabilities [25]. A p-value of 0.1 is considered statistically significant.

## Results

**Figure 1:**
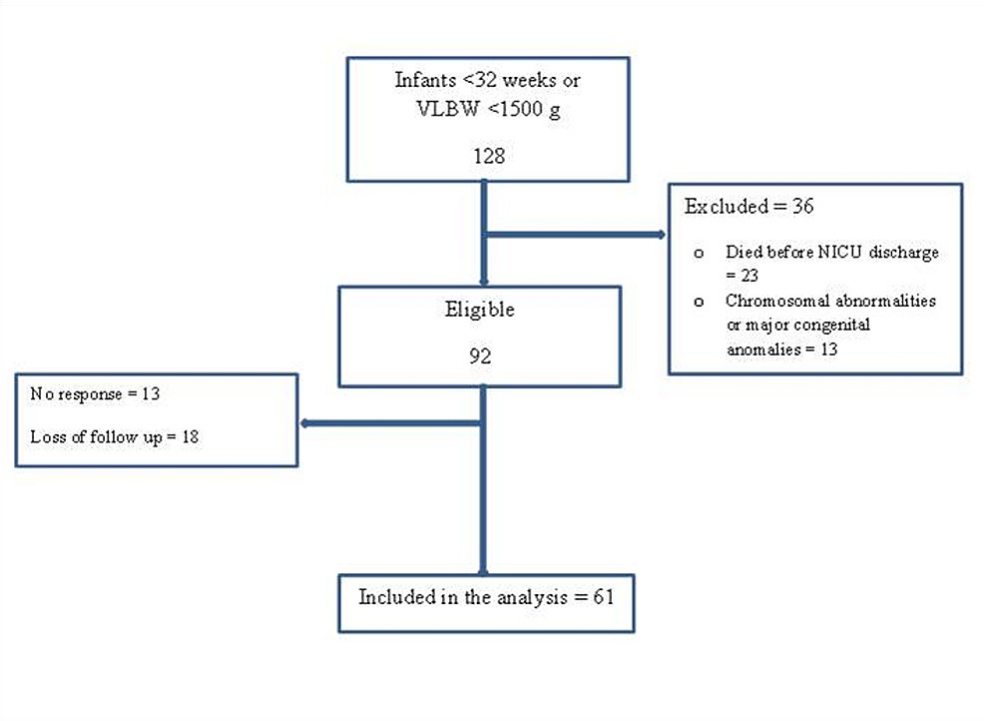
Patient's flow. VLBW: Very low birth weight; NICU: Neonatal intensive care units.

The study screened 128 preterm infants of less than 32 weeks gestational age or VLBW. Thirty-six preterm infants were excluded; 23 infants died before discharge, and 13 reported chromosomal abnormalities or major congenital anomalies. After exclusion, 92 infants were eligible; 13 did not respond, while 18 lost to follow-up either because they did not complete the online survey or exceeded the age limit due to the COVID-19 pandemic. There were no reported deaths after NICU discharge. Finally, 61 preterm infants completed sufficient assessment to be included in the study's primary outcomes (Figure 1). There were 18 (10%), 16 (26.2%), 12 (19.7%), and 23 (37.7%) who completed the assessment around 18, 20, 22, and 24 months corrected age, respectively.

Based on ASQ3-A, at the corrected age of 18-24 months; 35 infants (57.4%) had a normal neurodevelopmental screen, and 26 infants (42.6%) had at least NDD (<1SD) in one of the five domains which are communication skills, gross motor, fine motor, problem-solving skills, and personal-social skills.

A summary of the maternal and neonatal demographics of the final sample is shown in Tables 1 and 2, respectively. Of the maternal characteristics, only perinatal events (defined as any preterm premature rupture of the membranes [PPROM], antepartum haemorrhage [APH], or placental abruption) were statistically significant (4 [11] vs. 10 [39%] in children without vs. with NDI, respectively, p-value: 0.03). Of the neonatal characteristics, there were almost similar demographics in terms of gender: boys, 33 (54.1%) and girls, 28 (45.9%), gestational age at birth, and birth weight. During NICU stay, the included infants had similar morbidities except for the development of periventricular leukomalacia (PVL) (0 vs. 6 [23%], p-value: 0.03) (Table 2).

**Table 1:** Maternal characteristics. PPROM: Preterm premature rupture of the membranes; C-S: Caesarean section; APH: Antepartum haemorrhage. * P-value <0.05

Maternal characteristics	No NDD 35 n (%)	With NDD 26 n (%)
Antenatal care	21 (62)	19 (73)
Antenatal steroids	30 (86)	23 (88)
Hypertension	7 (20)	2 (8)
Preeclampsia	7 (20)	2 (8)
Diabetes	5 (14)	4 (5)
C-S	27 (77)	19 (73)
Perinatal events (PPROM, Abruptio, APH)	4 (11)	10 (39)*

**Table 2:** Neonatal characteristics and in-hospital morbidities. NDD: Neurodevelopmental delay; GA: Gestational age; BW: Birth weight; SGA: Small gestational age; IVH: Intraventricular hemorrhage; PVL: Periventricular leukomalacia; PDA: Hemodynamic significant patent ductus arteriosus; NEC: Necrotizing enterocolitis > stage I; ROP: Retinopathy of prematurity more than stage II or plus disease; BPD: Bronchopulmonary dysplasia with the requirement of oxygen by 28 days of life; MV: Mechanical ventilation. * Mean (SD)  ** Median (IQR)

Neonatal characteristics	No NDD 35 n (%)	With NDD 26 n (%)
Male	21 (60)	12 (46)
Female	14 (40)	14 (54)
GA mean (SD)	29.5 (2.1)	28.6 (2.6)*
GA =>28 weeks	27 (77)	16 (62)
GA <28 weeks	8 (23)	10 (39)
BW mean (SD)	1281 (311)	1150(401)
BW =>1000g	27 (77)	15 (58)
BW <1000g	8 (23)	11 (42)
Apgar Score at 5 min	8 (7-9)	8 (7-9)**
Cord pH	7.24 (0.08)	7.24 (0.09)*
SGA	3 (9)	0 (0)
Any IVH	4 (11)	5 (19)
IVH (>grade II)	0 (0)	4 (9.7)
Ventriculomegaly	1 (3)	0 (0)
Hydrocephalus	0 (0)	1 (4)
PVL	0 (0)	6 (23)
Sepsis	17 (49)	10(39)
PDA	13 (37)	9 (35)
NEC	1 (3)	4 (15)
ROP	0 (0)	2 (8)
Surgery	6 (17)	3 (12)
BPD	4 (11)	7 (27)
Postnatal steroids	3 (9)	5 (19)
Intubation	21 (60)	19 (73)
MV	11 (31)	6 (23)
Weight at discharge	1963 (451)	1969 (369)*
CGA at discharge	36 (35-39)	36 (35-39)**
LOS	37 (29-78)	57 (36-95)**

From a total score of 300, each domain accounts for 60 points. Out of 300, the lowest total score was 35, and the highest total score was 275. Children with a normal screen had a mean (SD) of 230 (31.2) compared to children with delay 137.3 (59), p-value <0.001. The delay in communication skills was detected in seven infants (11.5%), gross motor: seven infants (11.5%), fine motor: 12 infants (19.7%), problem-solving skills: twelve infants (19.7%), and personal-social skills: 14 infants (23%). In regards to the number of domains affected, in infants with delays, 15 (24.6%) had only one domain affected, while the rest 11 (18.%) had more than one domain affected. Of notice, all infants who had three or more domains affected corresponded to <1SD of the total score, while those who had all five domains affected corresponded to <2 SD. In univariate analysis, only perinatal events were statistically significant of the maternal factors 4 (11%) versus 10 (39%) in infants without NDD and with NDD, respectively. While in the analysis of neonatal factors, PVL was statistically significantly higher 0 (0%) versus 6 (23%) (Table 2). Using Firth’s logistic regression model that included the significant variable in the univariate analysis, PVL and perinatal events were independently significant predictors for NDD, where PVL coefficient: 2.9, p-value: 0.05 and perinatal events coefficient: 1.34, p-value: 0.04, penalized log-likelihood: -33.2 and Wald ch2: 7.5, p-value: 0.02.

## Discussion

This study provides information on the NDD rates of Saudi preterm, VLBW infants born in the KAMC-WR using the ASQ3-A screening tool in a cross-sectional design, accounting for 42.6%. The prevalence of NDD in preterm infants is well-documented, according to multiple studies [3,26,27].

Since ASQ3-A has a high negative predictive value, it would decrease the workload and the need for extensive neurodevelopmental testing. In addition, ASQ3-A is a cost-effective screening tool as it selects only smaller groups of high-risk children who require further assessment. Whereas a larger group of high-risk children like moderate and late preterm-born children, children with congenital defects or any other severe malformation who require neonatal surgery can be assisted by other essential available neurodevelopmental testing [21]. Moreover, a retrospective study compared Bayley and ASQ3, and they found no statistically significant difference in the performance of Bayley (81.1%) compared to ASQ3-A (83.3%). Furthermore, ASQ3-A had a kappa score of (k = 1.00). As a result, ASQ3 scores seemed satisfactory in conducting multidisciplinary team referral decisions and general proficiency in all situations [28]. As seen in this study, all 61 preterm infants initially recruited had complete demographic data with all morbidities, which is an excellent source to record the prevalence of common morbidities known to be associated with less than 32 weeks or VLBW preterm infants.

A study in France using ASQ3 on 224 very preterm born children at the corrected age of 2 reported that 27% of children had abnormal ASQ3 scores, whereas our study showed that 42.6% of the children had abnormal ASQ3-A scores. The difference could be explained by the low number of samples collected or the chances of high false-positive results [21]. A local retrospective study demonstrated that 14.2% of a total of 367 preterm infants aged 24-36 months had abnormal cognitive function according to Bayley Infant Neurodevelopmental Screener (BINS). Such screening tools are old and not widely used and are based on an older version of Bayley-II [17,29].

Compared to a prospective study done by Agarwal PK et al. to evaluate VLBW and preterm at 9,12,18,24 months without congenital malformation by using ASQ3, The NDI in 18 and 24 months in the overall domain was 43% which was comparable to our study, 42.6%. Moreover each domain was; communication accounted for 12.5% vs. 11.5%, gross motor 15% and 11.5%, fine motor 13.8% vs. 19.7%, problem-solving 12.5% vs. 19.7%, personal-social 14.1% and 23%, respectively. Such comparable figures are reassuring. Of note, they used 2SD below the mean as a cut-off for NDD; however, with sensitivity and specificity averaging 70%, and net present value (NPV) of 94% compared to Bayley-III, our approach of choosing 1SD is considered more conservative [30]. Such an approach in a country still lacking standardized definitive diagnostic psychomotor and cognitive testing is considered acceptable.

The study that was done in King Khalid University Hospital in Riyadh showed that the male gender is a significant predictor of poor outcomes. On the other hand, our study showed that NDD was almost comparable in males [29]. Such male predominance is known in the literature; however, our results could be affected because of high loss to follow-up. A study of children at 24 months showed that abnormal neurodevelopmental outcomes were more prominent in children with risk factors as pathologies of neonates, pregnancy or delivery, and birth before 37 weeks of gestation. A strong correlation was found between adverse neurodevelopmental outcomes and the combined effect of risk factors and neonatal pathologies, particularly complicated neonatal sepsis and CNS malformation [31,32]. This was no different from what we got as prenatal events and PVL were strong independent predictors for NDD.

In addition, a meta-analysis of 11 studies found that 18.6% of patients with isolated severe bilateral ventriculomegaly have mild/moderate disability, and 39.6% have a severe disability [33]. In Portugal, a study published in 2020 reported that severe neurodevelopmental deficit was diagnosed in 30.4% of premature infants born with severe peri-intraventricular hemorrhage. These studies support our results that concluded that CNS abnormalities, including IVH grade 3 and 4, ventriculomegaly, hydrocephalus, and periventricular leukomalacia, are major risk factors for NDD in preterm infants [34].

In our study, other risk factors were comparable between the groups, especially GA and BW, hence predictors were insignificant. However, a larger sample size could have more robust results that concur with the literature.

This study screened for more detailed domains related to NDI. Most of the other similar studies focused on either significant or fewer outcomes. In addition, ASQ3-A is a very reliable screening method to be used in limited settings. This study is the first study to use ASQ3-A to screen for NDI in Saudi Arabia; further validation studies using this tool in Arabic-speaking populations are required.

We collected data of 61 children, while a higher number of data is recommended to show more accurate results. Low infants' numbers could be explained by the delay caused by the COVID-19 pandemic. Also, this study is conducted in a single center, which might not reflect the actual prevalence in Saudi Arabia. We recommend a national multi-center study with multidomain factor assessment, including socioeconomic and antenatal variables [35,36]. The follow-up should be comprehensive, considering particular risks such preterm infants have, like increased risk of respiratory diseases and health care utilization, especially during the COVID-19 pandemic [37-39]. National guidelines should be developed based on country-specific resources and parents' values [36].

## Conclusions

This single-center study in Saudi Arabia screened preterm, VLBW infants based on ASQ3-A. Twenty infants (42.6%) had an abnormal NDD at a corrected age of 18-24 months. Perinatal events and PVL were independent predictors of NDD. We recommend that all preterm VLBW infants in Saudi Arabia be evaluated by a neurodevelopmental screening tool, ASQ3-A, especially in resource-limited settings to start early intervention. Also, more extensive multicenter studies are to be carried out with definitive diagnostic tools to have a national benchmark for the long-term NDI.

## References

[1] (2017). World Health Organization. Preterm birth. http://www.who.int/mediacentre/factsheets/fs363/en/.

[2] Quinn JA, Munoz FM, Gonik B (2016). Preterm birth: case definition & guidelines for data collection, analysis, and presentation of immunisation safety data. Vaccine.

[3] Rogers EE, Hintz SR (2016). Early neurodevelopmental outcomes of extremely preterm infants. Semin Perinatol.

[4] Moutquin J-M (2003). Classification and heterogeneity of preterm birth. BJOG.

[5] Low Birthweight | Children's Hospital of Philadelphia [Internet]. Chop.edu (2018). Children's Hospital of Philadelphia. Low Birthweight. https://www.chop.edu/conditions-diseases/low-birthweight.

[6] (2018). Children's Hospital of Philadelphia. Very Low Birthweight. http://www.chop.edu/conditions-diseases/very-low-birthweight.

[7] Very Low and Extremely Low Birthweight Infants [Internet]. UCSF Children's Hospital (2004). UCSF Children's Hospital. Very Low and Extremely Low Birthweight Infants. https://www.ucsfbenioffchildrens.org/-/media/project/ucsf/ucsf-bch/pdf/manuals/20_vlbw_elbw.pdf.

[8] (2017). What are the risk factors for preterm labor and birth?. https://www.nichd.nih.gov/health/topics/preterm/conditioninfo/who_risk.

[9] (2018). Mortality and acute complications in preterm infants. Preterm Birth: Causes, Consequences, and Prevention.

[10] Blencowe H, Lee AC, Cousens S (2013). Preterm birth-associated neurodevelopmental impairment estimates at regional and global levels for 2010. Pediatr Res.

[11] (2018). UK HealthCare. Short and Long-Term Effects of Preterm Birth. https://ukhealthcare.uky.edu/wellness-community/news-events/health-information/short-and-long-term-effects-preterm-birth.

[12] Ward RM, Beachy JC (2003). Neonatal complications following preterm birth. BJOG.

[13] (2018). Brighton Center for Pediatric Neurodevelopment. What is Neurodevelopment?. http://www.bcpn.org/what-is-neurodevelopment-.html.

[14] (2018). Patient.info. Premature Babies and their Problems. https://patient.info/doctor/premature-babies-and-their-problems.

[15] Soleimani F, Zaheri F, Abdi F (2014). Long-term neurodevelopmental outcomes after preterm birth. Iran Red Crescent Med J.

[16] Msall ME, Buck GM, Rogers BT, Merke D, Catanzaro NL, Zorn WA (1991). Risk factors for major neurodevelopmental impairments and need for special education resources in extremely premature infants. J Pediatr.

[17] Gücüyener K, Ergenekon E, Soysal AS, Aktaş A, Derinöz O, Koç E, Atalay Y (2006). Use of the bayley infant neurodevelopmental screener with premature infants. Brain Dev.

[18] Squires J, Bricker D (2009). Ages & Stages Questionnaires, Third Edition (ASQ- 3): A Parent Completed Child Monitoring System.

[19] Council on Children with Disabilities,  Section on Developmental Behavioral Pediatrics,  Bright Futures Steering Committee,  Medical Home Initiatives for Children with Special Needs Project Advisory Committee (2006). Identifying infants and young children with developmental disorders in the medical home: an algorithm for developmental surveillance and screening. Pediatrics.

[20] Squires J, Bricker D (2020). Ages & Stages Questionnaires®️ in Arabic, Third Edition (ASQ®️-3 Arabic) [Internet]. Products.brookespublishing.com. Ages & Stages Questionnaires®️ in Arabic, Third Edition (ASQ®️-3 Arabic): A Parent-Completed Child Monitoring System.

[21] Kerstjens JM, Nijhuis A, Hulzebos CV (2015). The ages and stages questionnaire and neurodevelopmental impairment in two-year-old preterm-born children. PLoS One.

[22] Simard MN, Luu TM, Gosselin J (2012). Concurrent validity of ages and stages questionnaires in preterm infants. Pediatrics.

[23] Schonhaut L, Armijo I, Schönstedt M, Alvarez J, Cordero M (2013). Validity of the ages and stages questionnaires in term and preterm infants. Pediatrics.

[24] (2021). Ages and Stages. Translations of ASQ. https://agesandstages.com/products-pricing/languages/.

[25] Puhr R, Heinze G, Nold M, Lusa L, Geroldinger A (2017). Firth's logistic regression with rare events: accurate effect estimates and predictions?. Stat Med.

[26] Kurimoto T, Ibara S, Kamitomo M (2021). Risk factors for mortality and neurodevelopmental impairment among neonates born at 22-23 weeks' gestation. Neonatology.

[27] Venkatesh KK, Leviton A, Hecht JL (2020). Histologic chorioamnionitis and risk of neurodevelopmental impairment at age 10 years among extremely preterm infants born before 28 weeks of gestation. Am J Obstet Gynecol.

[28] Mackin R, Ben Fadel N, Feberova J (2017). ASQ3 and/or the Bayley-III to support clinicians' decision making. PLoS One.

[29] Sobaih BH (2018). Long-term cognitive outcome of very low birth-weight Saudi preterm infants at the corrected age of 24-36 months. Saudi Med J.

[30] Agarwal PK, Shi L, Daniel LM (2017). Prospective evaluation of the Ages and Stages Questionnaire 3rd Edition in very-low-birthweight infants. Dev Med Child Neurol.

[31] Tskimanauri N, Khachapuridze N, Imnadze P, Chanadiri T, Bakhtadze S (2018). Correlation between perinatal risk factors and neurodevelopmental outcomes in children at 24 months of age. Georgian Med News.

[32] Rodríguez-Trujillo A, Ríos J, Ángeles MA (2019). Influence of perinatal inflammation on the neurodevelopmental outcome of premature infants. J Matern Fetal Neonatal Med.

[33] Carta S, Kaelin Agten A, Belcaro C, Bhide A (2018). Outcome of fetuses with prenatal diagnosis of isolated severe bilateral ventriculomegaly: systematic review and meta-analysis. Ultrasound Obstet Gynecol.

[34] Amaral J, Peixoto S, Faria D, Resende C, Taborda A (2020). Survival and neurodevelopmental outcomes of premature infants with severe peri-intraventricular hemorrhage at 24 months of age. Acta Med Port.

[35] Al-Hindi MY, Aljuhani H, Alnajjar AR, Alessa S, Alqurashi M, Faden YA (2020). Examining the association between parental socioeconomic status and preterm birth using multidomain social determinants scale in a tertiary care center in Saudi Arabia. Cureus.

[36] Al-Hindi MY, Al Sayari TA, Al Solami R (2020). Association of antenatal risk score with maternal and neonatal mortality and morbidity. Cureus.

[37] Alharbi AS, Alqwaiee M, Al-Hindi MY (2018). Bronchiolitis in children: the Saudi initiative of bronchiolitis diagnosis, management, and prevention (SIBRO). Ann Thorac Med.

[38] Al-Hindi MY, Alshamrani ZM, Alkhotani WA, Albassam AB, Amin Tashkandi AM, AlQurashi MA (2021). Utilization of health-care resources of preterm infants during their first 2 years of life after discharge from neonatal intensive care unit. J Clin Neonatol.

[39] Alharbi AS, Alzahrani M, Alodayani AN, Alhindi MY, Alharbi S, Alnemri A (2021). Saudi experts' recommendation for RSV prophylaxis in the era of COVID-19: consensus from the Saudi Pediatric Pulmonology Association. Saudi Med J.

